# Association between mutation of the *NF2* gene and monosomy 22 in menopausal women with sporadic meningiomas

**DOI:** 10.1186/1471-2350-14-114

**Published:** 2013-10-30

**Authors:** MariaDolores Tabernero, María Jara-Acevedo, Ana B Nieto, Arancha Rodríguez Caballero, Álvaro Otero, Pablo Sousa, Jesús Gonçalves, Patricia H Domingues, Alberto Orfao

**Affiliations:** 1 Instituto de Estudios Ciencias de la Salud de Castilla y León (IECSCYL), Soria, Spain; 2 Instituto de Investigación Biomédica de Salamanca (IBSAL), Salamanca, Spain; 3 Centre for Cancer Research (CIC), Instituto de Biología Molecular (IBMCC), Centro superior de Investigaciones Científicas (CSIC), Universidad de Salamanca (USAL), Salamanca, Spain; 4 Neurosurgery Service of the University Hospital of Salamanca, Salamanca, Spain

**Keywords:** Mutation, *NF2* gene, Sporadic meningiomas, Monosomy 22, Menopausal women

## Abstract

**Background:**

Meningioma was the first solid tumor shown to contain a recurrent genetic alteration e.g. monosomy 22/del(22q), *NF2* being the most relevant gene involved. Although monosomy 22/del(22q) is present in half of all meningiomas, and meningiomas frequently carry *NF2* mutations, no study has been reported so far in which both alterations are simultaneously assessed and correlated with the features of the disease.

**Methods:**

Here, we analyzed the frequency of both copy number changes involving chromosome 22 and *NF2* mutations in 20 sporadic meningiomas using high-density SNP-arrays, interphase-FISH and PCR techniques.

**Results:**

Our results show a significant frequency of *NF2* mutations (6/20 patients, 30%), most of which (5/6) had not been previously reported in sporadic meningiomas. *NF2* mutations involved five different exons and led to a truncated protein (p.Leu163CysfsX46, p.Phe62LeufsX61, p.Asp281MetfsX15, p.Phe285LeufsX11, p.Gln389ArgfsX37) and an in frame deletion of Phe119. Interestingly, all *NF2* mutated cases were menopausal women with monosomy 22 but not del(22q).

**Conclusions:**

These results confirm and extend on previous observations about the high frequency and heterogeneity of *NF2* mutations in sporadic meningiomas and indicate they could be restricted to a well-defined cytogenetic and clinical subgroup of menopausal women. Further studies in large series of patients are required to confirm our observations.

## Background

Meningioma was the first type of solid tumor which already in 1967, was shown to contain a specific recurrent genetic alteration consisting of loss of a chromosome 22 in around half of the cases [1]. The association observed between the dominantly-inherited neurofibromatosis type-2 syndrome and central nervous system (CNS) tumors such as meningiomas and schwannomas, has rapidly led to the identification of the NF2 gene located in chromosome 22q as a candidate predisposing gene in both familial and sporadic meningiomas [2,3]. However, loss of chromosome 22 and/or del(22q) are only found in a fraction of all meningiomas suggesting that molecular and chromosomal changes other than those targeting the *NF2* gene may also be involved in the development of meningiomas [4,5]. Since the earliest reports till 1994, more than 400 different *NF2* mutations have been described in meningiomas [6-27]; however, the precise significance of the *NF2* gene status in sporadic meningiomas remains unclear. Based on sequencing data from the literature, *NF2* mutations may involve all exons of the gene except exon 16. Interestingly, an association between *NF2* gene mutations and specific subtypes of meningiomas such as fibroblastic and transitional tumors has been observed [7-9,24], although such data could not be always confirmed in other series [28].

In this study, we report on the frequency of *NF2* mutations in a group of 20 sporadic WHO grade I/benign meningiomas and its association with the distinct genetic and histopathological subtypes of meningiomas. At the same time, we also review the literature reported so far on *NF2* mutations in meningiomas. Overall, six distinct *NF2* mutations were found (6/20 cases; 30%) five of which had not been previously reported in sporadic meningiomas [10,24,29]. Noteworthy, *NF2*-mutated tumors were systematically associated with complete loss of chromosome 22 (monosomy 22) but not del(22q), usually in the absence of additional genetic alterations involving other chromosomes as assessed by both interphase fluorescence in situ hybridization (iFISH) and single nucleotide polymorphism (SNP) arrays. These results, together with the higher median age and the slightly greater frequency of transitional meningiomas, among the mutated cases, suggest that *NF2*-mutated tumors may represent a uniquely well-defined subgroup of sporadic meningiomas.

## Methods

### Patients and samples

A total of 20 adult WHO grade I (sporadic) meningioma patients (3 males and 17 females; mean age of 60 ± 16 years) were included in this study. Prior to entering the study, informed consent was given by each individual and the study was approved by the ethics committee of the University Hospital of Salamanca (Salamanca, Spain). Classification of meningioma was established according to the WHO criteria, with the following distribution: meningothelial meningiomas, 10 cases; transitional, 7, and; fibroblastic meningiomas, 3 tumors. In each individual patient, paired fresh EDTA-anticoagulated peripheral blood (PB) and tumoral samples were obtained in parallel and stored in liquid nitrogen (−150°C), until analysed.

### DNA extraction

Both tumoral and PB cells’ DNA was extracted using the QIAamp DNA mini kit (Qiagen, Hilden, Germany) following the instructions of the manufacturer. A NanoDrop-1000 Spectrophotometer (Nano-Drop Technologies, Wilmington, DE, USA) was used to quantify the amount of DNA obtained and assess its quality.

### Copy number alterations and loss of heterozygosity (LOH) of chromosome 22 by iFISH and SNP-arrays

In order to confirm the presence of numerical changes involving chromosome 22, iFISH studies were performed with commercially available probes for chromosome 9 and 22q (LSI bcr/abl ES DC Probe). In addition, in 15/20 patients copy number alterations and LOH were analyzed with the GeneChip Human Mapping 250 K Nsp and 250 K Sty-arrays (Affymetrix), as previously reported [30].

### Identification of *NF2* mutations

DNA from the 20 meningioma samples was amplified by conventional PCR [20]. In order to identify mutations in the *NF2* gene sequence, 32 customized primers were used. Oligonucleotide primer sequences were obtained from the UniSTS database at NCBI (http://www.ncbi.nlm.nih.gov).

PCR products were analysed by capillary electrophoresis using an ABI 3130xl instrument (Applied Biosystems, Foster City, CA, USA) and the Chromas (Technelysium Pty Ltd*,*http://technelysium.com.au) software was used to analyze the DNA sequences obtained.

### Statistical analyses

The statistical significance of differences observed between groups was assessed by the Student T and the Mann–Whitney U tests, for parametric and non-parametric (continuous) variables, respectively; for qualitative variables, the *X*^*2*^ test was used (SPSS software, SPSS 15.0, SPSS Inc, Chicago, IL, USA). *P*-values <0.05 were considered to be associated with statistical significance.

## Results

### Frequency, localization and type of *NF2* mutations in sporadic meningiomas

**Figure 1:**
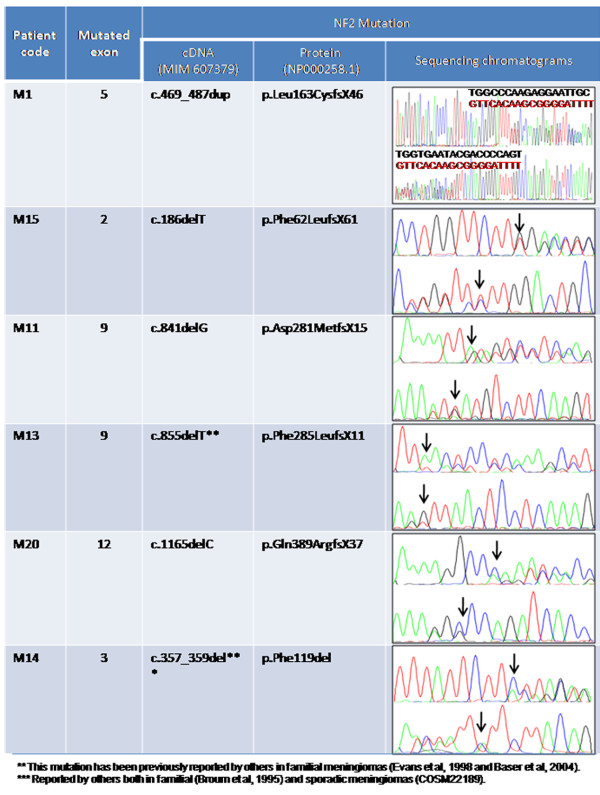
***NF2 *mutations.** Detailed description of the 6 *NF2* mutations (a 19 bp duplication and 5 deletions) identified among the 20 meningiomas analyzed, including the position of the alterations identified, the potential effect of each mutation and their sequencing chromatograms (the positions of the deleted base(s) are indicated with an arrow).

*NF2* gene mutations were found in 6/20 meningiomas studied (30%) (Table 1). Specific *NF2* mutations identified consisted of four deletions of a single base, one deletion of 3 bp and a duplication of 19 bp, (Figure 1). Therefore, changes in cDNA consisting of a c.186delT, c.841delG, c.855delT and c.1165delC were found in those four cases with single nucleotide deletions, such mutations generating the synthesis of the p.Phe62LeufsX61, p.Gln389ArgfsX37, p.Asp281MetfsX15 and p.Phe285LeufsX11 truncated proteins, respectively (Figure 1). The remaining deletion identified involved three consecutive bp (“CTT”) at exon 3 (c.357_359del) also leading to an in frame deletion of Phe119, and the duplication of 19 bp involved positions 469 to 487 of the *NF2* gene, leading to a p.Leu163Cys mutated nf2 protein with a stop after 46 codons (Figure 1).

**Table 1:** Clinical, histopathological and genetic characteristics of meningioma patients grouped according to the presence vs absence of NF2 gene mutations (n = 20)

**Patient features**		**NF2-mutated meningiomas**	**Non-mutated meningiomas**	**p-value**
		**(n = 6)**	**(n = 14)**	
Age (median in years)		73 (56–82)	53 (26–80)	0.03
Gender (Male/Female)		0/6	3/11	0.32
		(0%/100%)	(21%/79%)	
	Skull base	1 (17%)	7 (50%)	
Tumor localization	Convexity	1 (17%)	2 (14%)	0.37
	Tentorium	4 (66%)	4 (29%)	
	Spinal	0 (0%)	1 (7%)	
Histological subtype	Meningothelial	2 (33%)	8 (57%)	
	Transitional	3 (50%)	4 (29%)	NS
	Fibroblastic	1 (17%)	2 (14%)	
Chromosome 22 status by iFISH	Diploid	0 (0%)	9 (64%)	0.005
	del(22q)	0 (0%)	3 (22%)	
	Monosomy 22	6 (100%)	2 (14%)	
Cytogenetic subgroups	Diploid	0 (0%)	7 (50%)	0.03
	Isolated monosomy 22	4 (67%)	2 (14%)	
	Complex-karyotype: with monosomy 22	2 (33%)	1 (7%)	
	with del(22q)	0 (0%)	2 (14%)	
	Complex-karyotype w/o −22/del(22q)	0 (0%)	2 (14%)	

### Association between *NF2* mutations and other chromosomal changes

From the 20 meningiomas analyzed, 7 showed a diploid karyotype (35%), another 6 (30%) had an isolated loss of chromosome 22, two or more chromosomal changes including loss of chromosome 22q were found in another 5 cases –monosomy 22 in 3 (15%) and del(22q) in the other 2 patients (10%)– and two meningiomas displayed multiple chromosomal losses/gains in the absence of monosomy 22/del(22q) (n = 2; 10%) (Table 1).

Interestingly, all 6 *NF2*-mutated tumors carried monosomy 22, which was the only chromosomal alteration in 4/6 cases. Consequently, *NF2*-mutated meningiomas accounted for most cases associated with monosomy 22 (6/9; 67%), including cases with isolated monosomy 22 (4/6; 67%) or with monosomy 22 combined with other chromosomal alterations (2/6, 33%); conversely, among all other cases except three, which were either diploid for chromosome 22, carried del(22q) or had multiple chromosomal losses/gains in the absence of monosomy 22/del(22q), showed no *NF2* mutations (0/11; p = 0.03)(Table 1).

### Chromosome 22 copy number alterations and LOH by SNP-arrays

**Figure 2:**
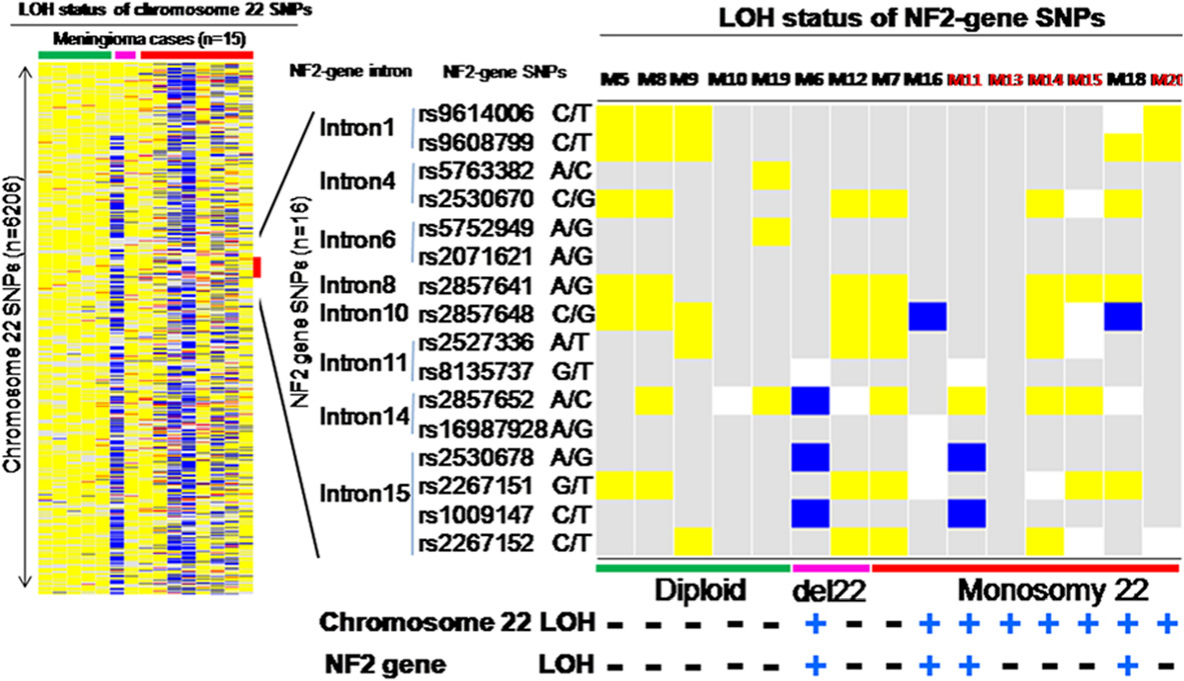
**LOH profiles of chromosome 22 and the *NF2 *gene in sporadic meningiomas (n = 15).** Rows (n = 6206) correspond to individual SNPs along the entire chromosome 22 and columns identify different meningiomas. Blue indicates presence of LOH, allele retention is shown in yellow, while red indicates conflict between PB DNA and tumor DNA SNP calls (AA or BB in PB and AB or BB and AB or AA in the tumor sample, respectively), and absence of call for non-informative SNPs (AA or BB) are depicted in white and grey, respectively. Two tumors (M7 and M12) showed a pattern compatible with biclonal allele deletions with copy number value of 1 in the absence of LOH. The *NF2* gene locus containing 16 SNPs in the array is amplified in the right side of the figure to better show the status of those SNPs heterozygously distributed in introns 1, 4, 6, 8, 10, 11, 14 and 15. LOH for three *NF2*-associated SNPs was found in one tumor (M6), for two SNPs in another case (M11) and for one SNP in another two meningiomas (M16 and M18).

As described above, iFISH showed losses of chromosome 22 in 11 cases; such losses consisted of monosomy 22 (9 cases; 45%) and del(22q) (2 cases; 10%) extending from the 18.207.392 bp position to the telomere of chromosome 22q (Figure 2 and Table 2). Noteworthy, LOH was investigated by SNP-arrays in 15/20 meningiomas, and it was found to involve chromosome 22 in 7/8 cases that had monosomy 22 and in 1/2 cases with del(22q). The number of chromosome 22 SNPs showing LOH varied between 3 and 920 SNPs. Among these patients, four SNPs (e.g., rs2857648, rs2857652, rs2530678 and rs1009147) were found to be associated with LOH for the *NF2* gene (Figure 2).

**Table 2:** Chromosome 22 status by iFISH vs SNP-arrays performed in 15/20 sporadic meningiomas

**Tumor**	**iFISH Karyotype**	Chromosome 22 status by SNP arrays* (n = 6206)			
**ID**		**Chromosome 22 status**	**Copy number values**	**N. of SNPs with LOH/N. of informative SNPs (%)**	
**M20**	−22q	−22	1.35 (1.01-1.93)	108	/ 1326 (8%)
**M18**	−22q	−22	1.19 (0.76-1.59)	365	/ 1131 (32%)
**M15**	Complex & -22q	−22	1.30 (0,85-2,03)	454	/ 1158 (39%)
**M14**	−22q	−22	1.34 (0.95-1.77)	80	/ 1472 (5%)
**M13**	−22q	−22	1.24 (0.92-1.8)	920	/ 1188 (77%)
**M11**	Complex & -22q	−22	1.20 (0.91-1.91)	694	/ 1043 (67%)
**M16**	−22q	−22	1.27 (0.81-1.75)	209	/ 1340 (16%)
**M7**	−22q,-Y	−22	1.52 (1.08-2.14)	18	/ 1505 (1%)
**M12**	Complex & del(22q)	del(22q11.22-qter)	1.50 (0.98-2)	19	/ 1683 (2%)
**M6**	Complex & del(22q)	del(22q11.22-qter)	1.34 (0.8-2.55)	763	/ 1195 (64%)
**M19**	Complex	Diploid	1.58 (1.12-2.06)	7	/ 1630 (0.4%)
**M10**	Complex	Diploid	1.71 (1.09-2.31)	9	/ 1770 (0.5%)
**M9**	Diploid	Diploid	1.75 (1.27-2.32)	3	/ 1712 (0.2%)
**M8**	Diploid	Diploid	1.92 (1.6-2.39)	5	/ 1681 (0.3%)
**M5**	Diploid	Diploid	1.79 (1.28-2.3)	3	/ 1696 (0.2%)

*LOH and copy number analyses involved 6206 SNP localized along the entire chromosome 22.

*iFISH* interphase fluorescence in situ hybridization, *SNP* single nucleotide polymorphism, *LOH* loss of heterozigosity.

### Association between *NF2* mutations and other features of the disease

Interestingly, all *NF2*-mutated meningiomas corresponded to female patients (6/6 vs 11/14, p > 0.05) with a higher median age vs all other cases (73 vs 53 years; p = 0.03)(Table 1). By contrast, a similar localization pattern was observed for the 6 *NF2*-mutated tumors and the other 14 non-mutated meningiomas (Table 1).

From the histopathological point of view, the 6 *NF2* mutated benign/grade I meningiomas showed a variable histology including 3 transitional meningiomas, two meningothelial tumors and one fibroblastic tumor, the frequency of transitional tumors being slightly higher than among non-mutated cases (3/6 vs 4/14 cases; p > 0.05) (Table 1).

## Discussion

Many studies have confirmed that loss of genetic material from chromosome 22 is by far the most frequent chromosomal alteration in meningiomas, being detected in around half of the cases. Such losses of chromosome 22 are heterogeneous and while they most frequently involve the whole chromosome (monosomy 22), in another substantial proportion of cases it consists of del(22q) with variable breakpoints, both alterations –monosomy 22 and del(22q) − being found either as the only chromosomal alteration or in combination with distinct alterations involving other chromosomes [30,31]. In parallel, multiple studies have also shown a variable frequency (between 14% and 78% of the cases) of *NF2* gene mutations in heterogeneous series of meningiomas consisting of between 12 to 170 patients [18,27], some of these reports specifically focusing on the investigation of particular subsets of patients such as recurrent meningiomas [6], rare histopathological subtypes [9] or patients affected by multiple meningiomas [12]. Recurrent chromosomal losses deviating from normal gene dosage present in tumor cells of various types, frequently point out the existence of one or more underlying tumor suppressor genes coded in the deleted DNA sequences. Because of this and the development of meningiomas in patients with neurofibromatosis carrying mutations of the *NF2* gene coded at chromosome 22q12.2, *NF2* has long been considered as the most relevant gene targeted by chromosome 22 losses in meningiomas [7,32]. However, careful analysis of the studies reported so far reveals that the association between chromosome 22 status and the presence of *NF2* mutations has not been investigated in detail in sporadic meningiomas.

In the present study, we demonstrate the existence of a close association between *NF2* mutation and monosomy 22 but not del(22q), in sporadic meningiomas. Whereas most cases with monosomy 22 showed *NF2* mutations, these were absent in all three patients who showed del(22q). Altogether, these findings suggest that the *NF2* gene would only be involved in the pathogenesis of a well-defined subset of all meningiomas, typically in association with monosomy 22. Of note, a few cases showed monosomy 22 in the absence of *NF2* mutations; these results support previous observations indicating that in meningiomas, monosomy 22 is typically found at higher frequencies than *NF2* mutations, although the frequency of both alterations also depends on the detection method. This, together with the observation that *NF2* mutations were always present in heterozygosis, could suggest that mutations of the *NF2* gene could be a secondary event in the development of meningiomas which would occur after the loss of chromosome 22 and that therefore, inactivation of other genes coded in chromosome 22 (e.g. *SMARCB1*[33]) could potentially play a major role in tumor development, even among *NF2* mutated cases. In line with this hypothesis, *NF2*- mutated meningiomas also showed a rather low incidence of LOH of the *NF2* gene by SNP-arrays. However, other mechanisms e.g. rearrangements identifiable by MPLA and other techniques, together with mutations in promoter and other non coding regions, and epigenetic mechanisms leading to inactivation of *NF2* gene expression, may also be involved and should be investigated to confirm or rule out this hypothesis. Of note, among cases with monosomy 22/del(22q) in the absence of *NF2* mutations, two patients showed lack of LOH, which could be related to the greater percentage of non-tumoral diploid cells present in both patients (data not shown) [34].

Despite all the above, detailed analysis of the clinical and histopathological features of the meningiomas patients studied at diagnosis, showed unique features for those sporadic tumors carrying *NF2*-mutations in association with monosomy 22. Accordingly, these cases systematically corresponded to older menopausal women, they more frequently showed a meningothelial histopathological appearance, and none of them had relapsed so far. These results suggest that *NF2*-mutated sporadic meningiomas may correspond to a unique cytogenetic and clinical subtype of meningiomas, which emerge at relatively advanced ages. In line with this hypothesis, previous studies also suggested the existence of a relationship between *NF2* gene mutations and the histology of meningiomas (e.g. transitional tumors [24]); however, this association remains controversial and in our series, the association between *NF2* mutated and transitional meningiomas did not reach statistical significance, in line with the findings of several groups [7,8,21].

Despite different mutations involving distinct exons of the *NF2* gene were found in each NF2-mutated meningioma case, they led to a truncated protein or a small in frame deletion and therefore, potentially also to a loss of (normal) function of the nf2 protein. Overall these findings are in line with the great heterogeneity of *NF2* mutations reported so far in the literature (Table 3). In fact, previous studies indicate that multiple distinct substitutions or deletions of one or more nucleotides may occur and that all *NF2* gene exons, except exon 16, may be involved (Table 3), exon 2 being the most frequently mutated (≈45% of all mutated tumors). Among our cases, deletions of one and three nucleotides together with duplication of a sequence of 20 nucleotides, were observed. Of note, with the exception of one mutation which had been previously described, none of the other five *NF2* gene mutations identified in our cases had been reported before in sporadic meningiomas (Table 3).

**Table 3:** Frequency of NF2-gene mutations in our patients and other series from the literature reporting >10 sporadic meningiomas

**Reference**	**No of mutated cases/total cases (%)**	**Exons involved**
Ruttledge, et al., 1994 [27]**	24/170 (14%)	2-7-8-9-10-11-12
LeKanne Deprez, et al., 1994 [26]**	19/48 (40%)	1-2-3-4-5-6-11-12
Merel, et al., 1995 [25]**	15/57 (26%)	1-2-3-4-5-6-7-8-9-12-13
Wellenreuther, et al., 1995 [24]	41/70 (59%)	1-2-3-4-5-6-7-8-10-11-12-13
Ng, et al., 1995 [23]	7/26 (27%)	4-5-6-7-10
Papi, et al., 1995 [22]**	9/61 (15%)	2-5-7-8-11
Harada, et al., 1996 [21]	8/23 (35%)	1-2-5-8-11-12
Ruttledge, et al., 1996 [20]	67/111 (60%)	1-2-3-4-6-7-8-10-11-12-13-14-15-17
De Vitis, et al., 1996 [29]	37/125 (30%)	2-3-4-5-6-7-8-11-12-13
Stangl, et al., 1997 [18]	10/12 (83%)	3-5-6-7-8-11-12
Leone, et al., 1999 [17]**	11/81 (14%)	2-7-11
Ueki, et al., 1999 [16]	10/50 (20%)	4-5-7-10-12-13
Evans, et al., 2001 [14]	4/27 (15%)	3-4-8-13
Joachim, et al., 2001 [13]*	26/61 (43%)	1-2-3-4-5-6-7-8-10-11-12-13
Szijan, et al., 2003 [11]	5/14 (35%)	2-3-8-12-13
Lomas, et al., 2005 [10]**	21/88 (24%)	1-2-3-4-7-11-12-13
Hartmann, et al., 2006 [9]^#^	21/80 (20%)	1-2-3-4-5-6-7-8-9-11-12-13-14-15
Kim, et al., 2006 [8]**	20/42 (48%)	1-2-4-5-7-10-11-12
Hansson, et al., 2007 [7]**	39/100 (39%)	1-2-3-4-6-7-8-9-10-12-13-14
Goutagny, et al., 2010 [6]^&^	14/18 (78%)	2-5-6-7-8-10-11-12-13
Tabernero et al. (2013)	6/20 (30%)	2-3-5-9-12
Total	414/1284 (32%)	1-2-3-4-5-6-7-8-9-10-11-12-13-14-15-17

^*^several radiation-induced meningiomas were included in this series; ^#^infrequent subtypes of meningiomas were analyzed in this series; ^&^meningioma cases showing progression were analyzed in this series.

^**^not all exones of the NF2 gene were investigated in this series.

## Conclusions

In the present study we confirm the relatively high frequency of distinct *NF2* mutations leading to a truncated protein in sporadic meningiomas, most of the identified genetic changes corresponding to mutations which had not been previously described. Interestingly, a clear association was found among our cases between mutation of the *NF2* gene and monosomy 22, but not del(22q); in addition, all *NF2*-mutated cases corresponded to older menopausal women, supporting the notion that *NF2*-mutated patients could represent a well-defined and unique cytogenetic and clinical subtype of meningiomas in which acquisition of monosomy 22 could be sequentially followed by *NF2* mutation during the development of the disease. Further studies in large series of meningioma patients are required to confirm these observations.

## Competing interests

We declare that we do not have received reimbursements, fees, funding, or salary from an organization that may in any way gain or lose financially from the publication of this manuscript, either now or in the future. In the past five years we only have received funding from an governamental research organization to finance this manuscript (the article processing charge) title “Association between mutation of the *NF2* gene and monosomy 22 in menopausal women with sporadic meningiomas.”

We do not hold any stocks or shares in an organization that may in any way gain or lose financially from the publication of this manuscript, either now or in the future.

We do not hold or applying for any patents relating to the content of the manuscript and we do not have received reimbursements, fees, funding, or salary from an organization that holds or has applied for patents relating to the content of the manuscript.

We do not have any other financial competing interests. There are not non-financial competing interests (political, personal, religious, ideological, academic, intellectual, commercial or any other) to declare in relation to this manuscript.

## Authors’ contributions

MDT conceived and design the study, analyzed/interpreted results and wrote the manuscript, MJA and ARC performed the mutation studies and analyzed/interpreted results, ABN performed the statistical and SNP-arrays analysis, AO, PS and JG provided the samples and clinical follow-up of the patients, PHD collected and performed the initial treatment of the samples and the iFISH studies, AOR participated in the design of the study, supervision, writing and revision of the final version of the manuscript. All authors read and approved the final manuscript.

## Pre-publication history

The pre-publication history for this paper can be accessed here:

http://www.biomedcentral.com/1471-2350/14/114/prepub
